# Supplementary material for: Towards the characterization of the hidden world of small proteins in Staphylococcus aureus, a proteogenomics approach
Source: PLoS Genet. 2021 Jun 1;17(6):e1009585. doi: 10.1371/journal.pgen.1009585 (PMC8195425; doi:10.1371/journal.pgen.1009585)
Supplement: S1 Table — (DOCX) [file pgen.1009585.s001.docx]

**S1 Table.** **All putative ORFs are classified using different criteria (if two ORF variants share the same class, the longer ORF is preferred).**

| **ORF class^1^** | **RBS class^1,2^** | **spacer class^1,3^** | **start codon class^1,4^** |
| --- | --- | --- | --- |
| 1 | 1 | 1 | 1 |
| 2 | 2 | 1 | 1 |
| 3 | 3 | 1 | 1 |
|  | | | |
| 4 | 1 | 1 | 2 |
| 5 | 2 | 1 | 2 |
| 6 | 3 | 1 | 2 |
|  | | | |
| 7 | 1 | 1 | 3 |
| 8 | 2 | 1 | 3 |
| 9 | 3 | 1 | 3 |
|  | | | |
| 10 | 1 | 2 | 1 |
| 11 | 2 | 2 | 1 |
| 12 | 3 | 2 | 1 |
|  | | | |
| 13 | 1 | 2 | 2 |
| 14 | 2 | 2 | 2 |
| 15 | 3 | 2 | 2 |
|  | | | |
| 16 | 1 | 2 | 1 |
| 17 | 2 | 2 | 2 |
| 18 | 3 | 2 | 3 |
|  | | | |
| 19 | - | - | 1 |
| 20 | - | - | 2 |
| 21 | - | - | 3 |

^1^ the lower the better

^2^ ribosomal binding sites (RBS) are classified according to their sequence [1]:

class 1: ideal sequence GGAGG

class 2: ideal sequence with one SNPs (G1T, G1C,

G1A, A3T, A3G, G5T, G5C or G5A)

class 3: ideal sequence with two SNPs (G1T, G1C,

G1A, A3T, A3G, G5T, G5C or G5A)

^3^ spacers between RBS and translation start are classified according to their length [1]:

class 1: more than 2bp

class 2: 1 or 2bp

^4^ start codons are classified according to their translation initiation activity [2]:

class 1: ATG, GTG, TTG

class 2: CTG, ATA, ATT, ATC

class 3: all other codons

1. McCarthy JE, Brimacombe R. Prokaryotic translation: the interactive pathway leading to initiation. Trends Genet. 1994;10(11):402-7.

2. Hecht A, Glasgow J, Jaschke PR, Bawazer LA, Munson MS, Cochran JR, et al. Measurements of translation initiation from all 64 codons in E. coli. Nucleic Acids Res. 2017;45(7):3615-26.
