# Supplementary material for: Towards the characterization of the hidden world of small proteins in Staphylococcus aureus, a proteogenomics approach
Source: PLoS Genet. 2021 Jun 1;17(6):e1009585. doi: 10.1371/journal.pgen.1009585 (PMC8195425; doi:10.1371/journal.pgen.1009585)
Supplement: S2 Table — (PDF) [file pgen.1009585.s002.pdf]

S2 Table. Selected output information provided by *Pepper*.

information	description
**PSM/Peptide level**	
peptideID	search-specific peptide IDs assigned by MaxQuant
protein accession	protein accession inferred by MaxQuant
MS/MS counts	sample-specific MS/MS counts
theoretic p*I*	estimated isoelectric point of the peptide
theoretic MW	estimated molecular weight of the peptide
theoretic GRAVY	estimated hydrophobicity of the peptide
total DNA matches	number of potential coding sites (DNA matches) in the given genome
**DNA match level**	
sequence	DNA sequence of the respective coding site
start	start coordinate in the given genome
end	end coordinate in the given genome sequence
length	length of the respective coding site
strand	coding strand
frame	reading frame
preceding aa	amino acid encoded by the codon directly preceding the potential coding site
match type	classification as existing annotation, extension of existing annotation, antisense to an existing annotation or novel
DNA level conservation	number of genomes of given collection(s) containing the identical DNA coding site
aa level conservation	number of genomes of given collection(s) encoding the identical peptide (allowing synonymous mutations)
in-frame overlapped genes	annotated genes spanning the coding site on the same strand and reading frame
out-frame overlapped genes	annotated CDS spanning the coding site but on a different strand or reading frame
up-/downstream distance	distance to the next up- or downstream CDS encoded on the same strand and reading frame
gene synteny	text-based representation of the gene synteny
**ORF level**[1]	
DNA sequence	DNA sequence of the respective ORF
aa sequence	translated sequence of the respective ORF
class	feature-based ORF class[2]
start	start coordinate in the given genome
end	end coordinate in the given genome
length	length of the putative ORF
strand	coding strand
frame	reading frame
annotation	existing annotation
start codon (class)	start codon and assigned start codon class[2]
RBS (class)	potential ribosomal binding site and assigned RBS class[2]
spacer (class)	spacer between RBS and translation start and assigned spacer class
in-frame overlapped genes	annotated genes spanning the ORF on the same strand and reading frame
out-frame overlapped genes	annotated CDS spanning the ORF but on a different strand or reading frame
up-/downstream distance	distance to the next up- or downstream CDS encoded on the same strand and reading frame
gene synteny	text-based representation of the gene synteny
supporting unique peptides	number of identified peptides supporting the respective ORF
DNA level conservation	number of genomes of given collection(s) containing the identical ORF
aa level conservation	number of genomes of given collection(s) encoding the identicval ORF product (allowing synonymous mutations)

information	description
upstream sequence	upstream sequence (21bp) preceding the respective ORF
theoretic p*I*	estimated isoelectric point of the ORF product
theoretic MW	estimated molecular weight of the ORF product
theoretic GRAVY	estimated hydrophobicity of the ORF product

[1] information of all potential ORF variants are listed

[2] see S1 Table S orf classes for details
