# Supplementary material for: Towards the characterization of the hidden world of small proteins in Staphylococcus aureus, a proteogenomics approach
Source: PLoS Genet. 2021 Jun 1;17(6):e1009585. doi: 10.1371/journal.pgen.1009585 (PMC8195425; doi:10.1371/journal.pgen.1009585)
Supplement: Attachment — Submitted filename: Reviewer comments_210505.docx [file pgen.1009585.s009.docx]

Dear Reviewers,

We are very grateful for your comments and suggestions that were very helpful to significantly improve our manuscript. In the following, we respond to each comment separately.

**Reviewer #1**

The authors have taken into account most of the suggestions of the referees, which have improved the manuscript. The work provides a catalog of novel small proteins that will require further analysis to proove their functional importance. Nevertheless this is certainly a useful study.

**Response**: Thank you very for this nice evaluation.

**Reviewer #2**

This is a revised version of the manuscript; Revealing the hidden world of small proteins in Staphylococcus aureus, a proteogenomics approach by Fuchs et al. In general, I find the authors’ replies to my satisfaction. I just have two remarks related to my previous comments:

**Response:** Thank you very for your assessment and for your helpful comments in this reviewing round.

1) One of my criticism was related to the limited innovative component of the workflow proposed in this work; proteomics + genomics (salt) validated by MS (pepper). The authors replied that Pepper is the component of innovation; MS data analysis for identification of small proteins, to crosscheck this data to ORF prediction. MS analysis provides a direct detection of small proteins whereas ribosome profiling provides indirect proof. Then, I do not understand why the authors validated MS data analysis with ribosome profiling. I suggest the authors made this innovative component more clear; maybe they can extend a bit the discussion section to explain the benefits of this technique in relation to other, already existing, approaches.

**Response:** In the discussion section (page 15, lines 519 to 529), we are now explaining more clearly what distinguishes our proteogenomics pipeline from already existing pipelines.

2) My second point was precisely related to pepper. sORFs embedded within other larger ORFs did not produce a reliable RFPs signal whereas other sORFs showed no translational activity. I meant to emphasize that, whereas this work represents an interesting starting point to identify small proteins in S. aureus, the pepper workflow seems to not provide a sufficiently strong experimental proof to validate that all sORF detected are bona fide small proteins. I understand this is beyond the scope of this work; this referee does not mean that the authors must check and validate all the small proteins detected and whether they play a biologically relevant role. Nonetheless, I think it would be a good idea to made a clear note, maybe in the discussion section, about the limitations of the pepper workflow. I think is important to discuss not only the sORF that were validated but also why other sORF were not possible to validate. This would make the workflow more robust to the eyes of the reader.

**Response:** We are now presenting more clearly the limitations of our proteogenomics pipeline regarding identification of SP100 by only one unique peptide and provide some alternative approaches that are very useful for validation of these proteins in future studies (page 16, lines 560 to 570).

**Reviewer #3**

With the clarification and inclusion of PSMs and criteria, which are indeed very high quality, I believe this manuscript well passes the bar for publication.

**Response:** We are very grateful for this evaluation.
