# Supplementary material for: Towards the characterization of the hidden world of small proteins in Staphylococcus aureus, a proteogenomics approach
Source: PLoS Genet. 2021 Jun 1;17(6):e1009585. doi: 10.1371/journal.pgen.1009585 (PMC8195425; doi:10.1371/journal.pgen.1009585)
Supplement: Attachment — Submitted filename: Reviewer comments.docx [file pgen.1009585.s008.docx]

Dear Reviewers,

We are very grateful for your comments and suggestions that were very helpful to significantly improve our manuscript. In the following we respond to each comment separately.

**Reviewer #1:**

This is an interesting paper, which presented a strategy combining computational approaches, proteomics, and ribosome profiling to gain knowledge on the missing part of the genome, the small proteins (< 100 aa) and peptides. The authors have made a rigorous study that will be useful for the community working on Staphylococcus aureus and in more general on bacteria. Their study revealed the existence of 178 small proteins and among them, 27 were not predicted by the genome annotation of the Newman strain, with 83% having a size above 50 aa. Many of these small proteins are basic suggesting that they might target nucleic acids or phospholipids. This work opens up many perspectives for further functional studies.

**Response:** Thank you very much for this nice evaluation.

I have only few comments to be addressed:

1- A significant number of the sORF encoding SP100 (almost 20%) does not present translational activity as demonstrated by the ribosome profiling data (Table S4). The sORF (below 100 nts) seems also to be poorly active as no translational activity was detected for almost 65% of them. As the authors mentioned, it is difficult to detect small proteins that are encoded inside other genes as they can be masked by the signal of the annotated gene. However, recent ribosome profiling improvements have been done to enrich ribosome density at the start codons (using for instance retapamulin to block translation initiation or Onc112 which blocks the entry at the elongation step). It would be of interest to mention these works that can improve the detection of small proteins below 50 aa.

**Response:** Thank you very much for this comment. Ribo-seq analysis in combination with retapamulin or Onc 112 treatment are an excellent approach to map translational start sites. They are well suited for the identification of novel sORFs and also for identification of internal translational start sites and detection of unusual start codons. Very comprehensive studies in *Escherichia coli* revealed putative internal start sites in a number of genes [see Meydan et al 2019, Weaver et al. 2019]. Hence, we initiated a ribosome profiling analysis in *S. aureus* in combination with retapamulin treatment. But it turned out that in *S. aureus* ribosomes were not efficiently blocked at the initiation step, possibly due to an inefficient transport of retapamulin into the cell. In the future, it will thus be of substantial importance to find similar substances stalling the initiation complex that work in *S. aureus* in order to provide an alternative method for a high-confidence prediction of novel small proteins in particular of those encoded within larger ORFs in this pathogen.

This aspect was also included in the manuscript (lines 464 to 466, lines 488 to 492).

The authors should better mention the limits of the proteomic approach they used. For instance, because they have used cytosolic protein fraction for proteomics, it might be possible that some of the peptides have been missed because they are either secreted or transported to the membranes.

**Response:** This is indeed an important point and is now acknowledged in the revised manuscript (lines 427 to 432). The focus of the present study was on the development of a proteogenomics workflow enabling us to identify not only annotated small proteins but also those that are as of yet missed in genome annotations. We used cytosolic protein extracts of *S. aureus* as reference as it is the most complex protein fraction in *S. aureus* and several protocols for proteolytic digestion, protein/peptide prefractionation and MS based peptide identification for this protein fraction are well established. MS data obtained by the different experimental approaches applied here provided an excellent basis for the validation of PEPPER, a bioinformatics workflow for bacterial proteogenomics facilitating not only the identification of high quality PSMs but also the assignment of these peptides to so far missed protein-coding regions of the genome and the prediction of novel sORFs. This workflow is now highly optimized and can be used for other protein fractions such as extracellular and membrane proteins. However, for the identification of SP100 in these protein fractions it will be essential to optimize the experimental workflow in advance. As described in previous publications (e.g. Omasits et al Genome Res 2013), large effort has to be undertaken in order to describe complete expressed proteomes including the membrane proteome. This was beyond the scope of this study.

3- The annotation of Table S4 can be significantly improved. For instance, I think that CspA protein is an RNA chaperone that is primarily involved in post-transcriptional and translational controls but not in transcriptional control. Most of the highly abundant SP100 are factors involved in ribosome function and biogenesis. Several of these genes are given as they belong to several bacterial ribosomal proteins. The authors should precise the meaningful. Does it mean that these sORFs are pseudogenes? It is also mentioned that the ORFSaNew0084 is an RNA binding protein, which binds to sRNAs and mRNAs, it is Hfq protein? The legend of Table S4 should be detailed (ORF type?).

**Response**: Information about the function of annotated SP100 provided in S4 Table was extended, re-organized and improved, e.g. by manual corrections of eggNOG functions for some proteins using information from other databases, from the literature and by adding the NCBI annotation (predicted with a local installation of the Prokaryotic Genome Annotation Pipeline of the NCBI) including the gene name. Hence, ORFSaNew0084 is now correctly listed as the Hfq protein. Several of the identified sORFs encode ribosomal proteins. The addition of the NCBI annotation makes it now clearer which specific ribosomal proteins are encoded by the here identified sORFs. None of these genes are pseudogenes.

Notably, we added a comprehensive additional sheet entitled “legend” in S4 Table, which explains the information provided in each column as well as the possible values (e.g. ORF-type and start codon class).

4- Page 13: the authors have mentioned that the sORFs they have identified are classified into three categories: sORFs in intergenic regions, sORFs overlapping with the 5' or 3' UTR of different ORFs, and located within a larger ORF but in a different reading frames. It would be of interest to add this information in Table S4 as well as the sequences of the proteins. Did they find sORFs as possible upstream leaders of other genes? Certainly that further work help to decipher the sORFs that are translated within operons from those that specifically control the translation of the downstream gene.

**Response:** Localization for the identified sORFs is indicated by the ORF-type, which is now presented much more clearly in S4 Table and described in the legend sheet. Moreover, information about the identified SP100 such as protein sequences, protein length, gene names and gene descriptions (NCBI’s PGAP; see point 3) were now included in S4 Table.

We identified several sORFs located proximal in sequence space to upstream or downstream genes encoding well characterized larger proteins with enzymatic activity such as aminopeptidase (NWMN_RS104440), glutamine amidotransferase (NWMN_RS10105), glycosyltransferase (NWMN_RS05075), ribonuclease J (NWMN_RS05355), cardiolipin synthase (NWMN_RS06950), and GTP- and ATP-binding proteins (NWMN_RS02350, NWMN_RS01520, NWMN_RS10370) involved in heme biosynthesis or DNA replication. Further functional analyses will reveal whether translation of these sORFs directly affects translational activity of the downstream genes, or whether these sORF products might modulate the activity of these proteins.

This aspect is now also discussed in the manuscript (lines 571 to 582).

5- The title has to be changed because even if the strategy is powerful, many of the small ORFs (below 50 aa) were not identified here... I would suggest something like "Towards the charactrization of the hidden world of small proteins..."

**Response:** We agree (also with respect to the focus on cytoplasmic proteins in this proof of principle study) with the reviewer and changed the title accordingly.

**Reviewer #2**

In the report, Revealing the hidden world of small proteins in Staphylococcus aureus, a proteogenomics approach, Fuchs et al., developed a bioinformatics workflow to identify potential small protein candidates in the genome of Staphylococcus aureus. The workflow combines proteomics and genomic data analysis and is validated by ribosome profiling. The approach is already established; comparable workflows (with certain variations from lab to lab) are described to explore the existence of small proteins in different organisms (e.g. Yang 211, Miravet-verde 2019; Weaver 2019, Venturini 2020). In this regard, the workflow is solid but it does not have a strong component of innovation away from the field of Staphylococcus aureus. Indeed, for S. aureus laboratories will be an interesting tool.

**Resonse:** The reviewer is right. Especially in recent years, various workflows were established aiming at the identification of small proteins that have not been considered by conventional genome annotations. Regarding sORF identification, the most important feature of the present approach is a fully automated MS data analysis executed by our bacterial proteogenomics pipeline *Pepper,* which provides empiric and evidence-based gene annotations. This pipeline automatically selects high quality PSMs for peptide identification and provides an algorithm for ORF prediction in bacteria based on identified peptides, which is unique. It is thus optimized for the identification of expressed SP100 not only in *S. aureus* but also in bacteria in general even by one unique peptide (as long as sufficient high-quality spectral information is available), and completely independent of existing genome annotations. Compared to highly sensitive ribosomal profiling approaches which are more frequently used for sORF identification, highly confident MS based identification of proteins provides direct evidence for the existence of sORF encoded proteins and validates not only translation of sORFs but also stability of their gene products.

The experimental part combines MS peptidic data coupled to ribosome profiling. The ribo seq experiment detected a number of ribosome-protected mRNA fragments (RPF) that were mapped to 76% of the total putative sORFs in the genome. However, only a few of them were validated with the corresponding MS peptidic signal. sORFs that are embedded within other larger ORFs did not produce a reliable RFPs signal while other sORFs showed no translational activity. Although this integrative work represents an interesting starting point for identifying small proteins in S. aureus, the strength of the experimental dataset does not allow to answer important questions as to whether these peptidic fragments detected by MS are bona fide small proteins and more importantly, whether they have any biological relevance or there is any indication that these peptides play a role in any cellular process (e.g. interaction with other proteins, phenotypes associated with their expression or virulence potential).

**Response:** This is a proof concept methodological study focusing on the reliable identification of sORFs in *S. aureus* using MS based techniques. This was very challenging since there was as yet little experience in the MS based identification of small proteins in particular of those proteins smaller than 50 aa. Therefore, the suitability of different parameters for a highly confident identification of small proteins, used by our proteogenomics workflow, had to be checked carefully. As a result, we present here a first comprehensive resource of expressed SP100 in *S. aureus*. Functional characterization of the identified SP100 (including phenotypic analyses of deletion mutants, identification of interaction partners) is indeed also highly relevant but beyond the scope of the present proof of principle study. However, this will be the focus of several follow up studies.

The authors obtained MS-based peptidic signal using gel-based and gel-free pre-fractionation methods and compared the two methods for their precision and accuracy to detect small proteins. It is unclear to this referee what is the purpose of this comparison, what are the benefits of one approach in comparison to the other and more importantly, what is the final conclusion of the comparative study. I understood that the authors used these two methods to detect common peptidic signals and used them as trustable signals. So, it seems that one must run the two approaches to obtained a reliable signal to detect small proteins.

**Response:** Our intention was to test the suitability of the different approaches for identification of SP100 in *S. aureus* using our newly developed proteogenomics workflow. At the end, we realized that the here applied proteomics methods and MS instruments were equally well suited for identification of small proteins in *S. aureus*. Each method identified a large set of common SP100, but also a significant number of unique SP100. From this data we concluded that the here applied methods complement each other and multiple approaches have to be applied to identify the entire set of SP100 in a given sample of soluble proteins. The same applies for the membrane or extracellular protein fraction and has to be specifically tested for these protein fractions. In addition, we could show that endoproteases selected for digestion of proteins significantly influenced the quality of SP100 identification. While we concluded that trypsin is favorable for identification of soluble small proteins in *S. aureus*, Bartel and coworker (2020) recommended Lys-C and Arg-C for identification of soluble small proteins in *Bacillus subtilis*.

These aspects are also addressed in the manuscript (lines 374 to 387, lines 521 to 531).

Other comments:

1) Regarding ribosomal profiling, this technique does not corroborate the existence of the identified SP100 as this technique operates at the mRNA level. Please rewrite this sentence acordingly.

**Response:** The sentence was modified according to the reviewer´s suggestion (lines 477 to 478).

2) The number and classification of the MS data is hard to follow. I had problems to understand this part of the manuscript. For instance, there are several values of SP100 found using different approaches/sections and is at times difficult to digest.

**Response:** According to the reviewer´s suggestions, we rigorously revised and re-wrote this section of the manuscript.

3) Why do the authors have focused on the strain Newman? It seems that this clinical isolate is not an MRSA and does not cause important infections these days.

**Response**: *S. aureus* Newman was selected as a model strain for the identification of SP100 in *S. aureus* for several reasons: (i) it is one of the sequenced *S. aureus* strains whose genome sequence is very well annotated, (ii) it is characterized by a relatively stable phenotype in different infection models (iii) it is simpler to genetically manipulate than MRSA strains (e.g. the COL strain) which is important for follow-up studies focusing on the functional characterization of SP100, (iv) its genome sequence codes for four prophages, which play a significant role in *S. aureus* virulence, (v) different proteomics protocols are well established for this strain and (vi) a huge number of MS based proteomics data are available that can be reanalyzed using our well established proteogenomcs pipeline, making this a very valuable resource for the community.

**Reviewer #3**

The authors report proteomic re-annotation of the S. aureus genome, with a combined LC-MS/MS and ribosome profiling approach leading to identification of 27 previously unannotated gene products <100 amino acids. Given the mushrooming number of recent reports of proteogenomic analysis of small proteins, including many bacteria, novelty of new studies is an important consideration. To that point, this paper most importantly offers (1) the first proteogenomic analysis of S. aureus Newman, an important pathogen, and (2) the development of a web interface for 6-frame genome translation database construction for MaxQuant searches. While many methods for 6-frame genomic databases have been previously reported, it seems worthwhile to create an accessible tool for non-specialists. In another more technical note, the application of multienzyme digest in proteogenomics has been underexplored and the demonstration that this approach (modestly) improves small protein detection in this study may be of some interest to proteomics practitioners. Other mass spectrometry methods reported, such as caparison of gel vs. non-gel-based prefractionation, are in line with previous reports using human samples (such as https://pubs.acs.org/doi/abs/10.1021/acs.analchem.6b00191 <https://pubs.acs.org/doi/abs/10.1021/acs.analchem.6b00191> ).

**Response:** We are grateful for this nice evaluation and very pleased that our open source tools SALT and Pepper, which are in combination powerful for highly automated proteogeniomcs studies with the special focus on the identification of novel sORFs in bacteria, are very well received. As recommended, additional references using different proteomics approaches to identify small proteins were included (lines 382 to 387, lines 503 to 509)

Major concern: It is true that many proteogenomics studies rely on single peptide-spectral matches (PSMs) for small protein identification, due to the low occurrence of unique tryptic peptides within short sequences. This, in itself, is not a concern. However, it is best practice to provide the unique PSMs, along with scores, mass errors, etc., for examination in the case of single peptide identifications - for example, see http://pubsapp.acs.org/paragonplus/submission/jprobs/jprobs_proteomics_guidelines.pdf <http://pubsapp.acs.org/paragonplus/submission/jprobs/jprobs_proteomics_guidelines.pdf> ?. This is particularly important for proteins reported for the first time. I may simply have missed this - in which case, I apologize - but I was unable to access the PRIDE data or find a reviewer key, nor was I able to locate reproduced PSMs in the supplementary material. While the authors have made clear that they utilized stringent criteria, manual inspection is still required.

**Response**: We appreciate this highly relevant comment. The required data for unique PSMs were uploaded to PRIDE and we apologize for the difficulties to get access to the data. The mass spectrometry proteomics data have been deposited to the ProteomeXchange Consortium (http://proteomecentral.proteomexchange.org) via the PRIDE partner repository with the dataset identifier PXD017932 (https://www.ebi.ac.uk/pride/archive username: reviewer60192@ebi.ac.uk; password: 4FpFaITj (select “profile” and “Review Submission”)).

For identification of SP100 by at least one unique peptide, we defined filter criteria based on a very comprehensive visual inspection of MS/MS spectra by experts. We required that there be a sequence tag of at least five consecutive b or y fragment ions or two times four consecutive b or y ions in a spectrum. In addition, we suggested that peptide specific mass tracks should have significant levels above the background levels. For automatic selection of PSMs that fulfill these specific requirements, the following filter criteria were applied by PEPPER: at least five consecutive b or y fragment ions or two times four consecutive b or y ions in a spectrum, the intensity coverage should be above 0.1, the Andromeda Score at least ≥ 40 and the posterior error probability no more than 0.1. These aspects, which were not sufficiently clear in the original manuscript, were now added revised manuscript (lines 299 to 310, lines 354 to 357)

Furthermore, spectra for PSMs which passed our filter criteria and were assigned to SP100 using the different MS based approaches are now also provided in the Supplemental Material. For SP100 identified by one unique peptide the number of MS/MS scans is now displayed in S5 Table providing evidence about the abundance of these proteins. Accordingly, 34 out of these peptides were identified in more than one experimental approach and 21 by at least 10 MS/MS scans in at least one experimental approach (Table S5). This important information was also included in the manuscript (lines 535 to 540).

In addition, we created peptide spectral libraries of all identified peptides for each experiment using Skyline (version 20.2.0.343). These spectral libraries are available at <https://cloudstorage.tu-braunschweig.de/getlink/fiPyaXFgHTDBVHromF4tS31L/> during the reviewing process.

Minor concern:

Pages 13-14: "For the latter two we can additionally distinguish between those located at the same strand and those located at the complementary strand. The majority (n=13) belongs to the group (iii) of which five are localized at the same strand (Fig 9). This group of proteins is highly interesting as their existence has been ignored for a long time and highlights the power of an unbiased proteogenomics approach as applied here."

This sentence seems to imply that overlapping same-strand genes have not yet been reported, which is not true and references should be provided. There is certainly precedent for their existence - at least in E. coli and human. For example, https://www.ncbi.nlm.nih.gov/pmc/articles/PMC5761644/ <https://www.ncbi.nlm.nih.gov/pmc/articles/PMC5761644/> , https://academic.oup.com/nar/article/48/3/1029/5556081 <https://academic.oup.com/nar/article/48/3/1029/5556081> , https://academic.oup.com/nar/article/48/3/1029/5556081 <https://academic.oup.com/nar/article/48/3/1029/5556081> , https://www.embopress.org/doi/full/10.15252/embr.202050640 <https://www.embopress.org/doi/full/10.15252/embr.202050640> , just to provide a few examples out of many.

**Response:** We apologize for the misunderstanding and modified the paragraph in the manuscript accordingly (lines 462 to 466).
